# The time has come for a UK-wide menopause education and support programme: InTune

**DOI:** 10.1177/17455057241277535

**Published:** 2024-09-12

**Authors:** Joyce Harper, Nicky Keay, Florence Rowe, Polly Van Alstyne, Shema Tariq

**Affiliations:** 1 Institute for Women’s Health, University College London, London, UK; 2 Division of Medicine, University College London, London, UK; 3 Business and Innovation Partnerships, University College London, London, UK; 4 Research Department of Infection and Population Health, Institute for Global Health, University College London, London, UK

**Keywords:** perimenopause, education, symptoms, women’s health, menopause

‘There are some different narratives out there around menopause and around things like HRT. And it does get quite controversial. And it is very, very confusing for a lot of women and people out there who are getting misinformation’. Focus group participant.

Our research at University College London (UCL) has identified a significant unmet need for menopause education and support.^1–5^ For example, a survey of over 6000 women revealed that only 2% of women feel they were well-informed about menopause prior to entering perimenopause, with nearly two-thirds reporting they had not been informed at all.^1^ Nearly 70% of women reported looking for information once they started experiencing symptoms; 17% of them waited until they had experienced symptoms to first search for information.^1^ Women reported accessing information from general practitioners, online including society web sites and through friends.^1,2^ However, the recent proliferation of menopause-related information (and misinformation) makes it harder to identify accurate and trusted sources. This leaves people entering this key life stage with a lack of information and support. It is therefore unsurprising that only 39% of women are accepting of approaching menopause, with a third reporting that they “dread” it.

We recognise that there is a gap in menopause education and support at a national level, and we want to address this. Our team at UCL comprises cisgender women of diverse ethnicities. Most of us have clinical and/or research experience in menopause, as well as personal lived experience of menopause. We all share a commitment to translating our research into interventions that can transform the lives of people experiencing menopause in the United Kingdom and beyond.

The National Institute of Health Research-funded PRIME study (Positive Transitions Through the Menopause), led by Dr. Tariq, investigated the impact of menopause on women with human immunodeficiency virus (HIV) in nearly 900 participants across England aged 45–60.^6^ PRIME highlighted a high prevalence of menopausal symptoms in women living with HIV, with limited sources of support and low levels of menopause-related health literacy.^7–9^ In response, three major UK HIV charities (NAM AIDSMap, Positively UK and Sophia Forum), in collaboration with Dr. Tariq, developed the first ever tailored education and peer support programme for women ageing with HIV. This programme has been led by and co-designed with women living with HIV and includes a series of information videos and a bespoke training programme for women living with HIV to become peer mentors, and therefore qualified to support other women getting older with HIV (GROWS, Growing Older Wiser and Stronger).^10^

Findings and learning from our survey research and the development of targeted peer support and education for women with HIV now inform our response to the recognised gap in menopause education and support at a general population level. We are developing InTune (The UnIted KiNgdom’s NaTional menopaUse educatioN and support programmE), the UK’s first ever national menopause education and support programme. Such a programme is long overdue and is likely to have positive, multidimensional impacts on women and people experiencing menopause, including improved quality of life and mental health. We anticipate that this programme will reduce healthcare utilisation, resulting in cost-savings to the health service.

We have a bold and ambitious vision to provide evidence-based, accessible and low-cost menopause education and peer-support across the United Kingdom. We want to give the right information, to the right individual, at the right time. We recognise the diversity of communities transitioning through the menopause and the importance of their unique needs being met. Our work so far highlights that any such programme will need to be flexible in mode of delivery and adapted in language and content for different audiences.

Through establishing key stakeholder support and partnerships, drawing upon our advisory board and implementing a rigorous co-design process (comprising stakeholder workshops, focus group discussion and a public consultation survey), we have strived to facilitate the meaningful exchange of knowledge and experience across a variety of stakeholders in the menopause space. We have established an advisory board who guide the development of the programme and provide access to networks (Table 1). Furthermore, we have secured partnerships with key charities: Wellbeing of Women (https://www.wellbeingofwomen.org.uk/) and the Sophia Forum (https://sophiaforum.net/), and we have support from the British Menopause Society (https://thebms.org.uk/) and the Royal College of Obstetricians and Gynaecologists (https://www.rcog.org.uk/).

**Table 1.** The UK Menopause Education and Support Programme Advisory Committee.

The UCL team: Joyce Harper Nicky Keay Shema Tariq Florrie Rowe Polly Van Alstyne Advisory Committee: Karen Arthur Carolyn Harris MP Geeta Kumar Rachel Lankester Janet Lindsay Annice Mukherjee Ann O’Neill Lesley Regan Alice Smellie Sophie Strachan Vikram Talaulikar Helen Tomlinson Sue Mann

UCL: University College London.

Co-design is central to our practice. We believe that individuals who were born female are experts. Co-designing the programme with our target audiences will ensure it is relevant, accessible and acceptable.^11,12^ A central organising principle of our programme is inclusion. It is important that InTune (and components offered within it) reaches marginalised groups so that existing health inequities are not inadvertently amplified. We recognise that experiences of menopause are highly individual, and we want to ensure that any education and support we develop takes into accounts the needs and preferences of who are disabled, racially minoritised, sexually minoritised and/or gender diverse, as well as those who have experienced early menopause and/or iatrogenic menopause.

Our co-design process has included 2 workshops (in October 2023 and July 2024), 12 focus group discussions and a public consultation survey (conducted in January to March 2024 via an online survey; focus groups and survey approved by UCL research ethics committee, reference: 9831/012). Following our first workshop comprising 57 key stakeholders including academics, clinicians, charity representatives, activists and other professionals working in menopause, for example, yoga practitioners, coaches and nutritionists, we drafted an outline syllabus which was refined in workshop 2 (Table 2). Our focus group discussions and survey data will enable further refining of programme design and content to ensure it meets the needs of a diverse range of people.

**Table 2.** The curriculum for the Perimenopause Programme.

1. What is menopause: definitions, age, and hormonal, biological, emotional and social changes 2. Symptoms: changes in periods, vasomotor symptoms, genitourinary symptoms, psychological symptoms 3. Complex menopause: such as early menopause, iatrogenic menopause, co-existing long term conditions 4. Diagnosing menopause: why we do not recommend a test if over the age of 45 5. Medical management: HRT (hormone replacement therapy), CBT (cognitive behavioural therapy), alternative therapies, other drugs (including new drugs) 6. Wellbeing: 1. Nutrition 2. Exercise 3. Sleep 4. Mental health (or psychological health) 5. Sex (including contraception) 6. Friendships and community 7. Positive aspects of menopause 8. Post-menopause: support to go away with and build upon

Our work so far has established that there is a need for two separate but interrelated programmes: Be Prepared for Menopause and the Perimenopause Programme.

We know that those who have not reached perimenopause are currently underserved and often do not recognise that they are experiencing perimenopausal symptoms, preventing care-seeking and access to support:

The lack of information is shocking, I didn’t know the symptoms so didn’t join the dots until I spoke to a friend. We are both nurses, and we didn’t know!

Survey participant#438.^1^

Be Prepared for Menopause will include an overview of menopausal symptoms and management *for* individuals who have not yet reached perimenopause, allowing them to be aware of symptoms, and will include the importance of lifestyle optimisation. We anticipate that this will reduce visits to primary care and improve individual quality of life. A prototype of Be Prepared for Menopause has been developed, and we will be piloting this with diverse audiences from 2024.

Our second programme is for individuals experiencing menopause-related symptoms. Inspired by models of antenatal classes, we will develop and deliver a 6–8 week group course. We hope this will be delivered within communities and through employers, fusing education, coaching and peer support. This programme is currently under development.

InTune will be flexible and adaptable, designed to meet people where they are at. We hope to offer both programmes in-person and online, adapting content and delivery for a diverse range of target audiences, including individuals who are neurodivergent, racially minoritised, gender-diverse, sexually minoritised, living with an existing health condition and/or have experienced premature or iatrogenic menopause. We now plan to secure further funding to refine our programme and ensure InTune is robustly assessed using the Medical Research Council’s Framework for Developing and Evaluating Complex Interventions.^13^

In summary, our previous research has highlighted an urgent need for accessible, evidence-based menopause education and support. We now wish to use our research expertise to respond to this. Our vision is of high-quality, evidence-based, inclusive menopause awareness, education and support, for everyone. We will achieve this by developing and delivering a non-commercial programme of holistic support and education about menopause, co-designed with stakeholders and the public. We believe that the time is right for In Tune, a national programme that will allow people to be In Tune with menopause, In Tune with their bodies and In Tune with each other.

For further information – visit www.ucl.ac.uk/global-health/uk-national-menopause-education-and-support-programme

## Declarations

**Ethics approval and consent to participate:** The focus groups and survey were approved by UCL Research Ethics Committee 9831/012.

**Consent for publication:** Not applicable.

**Availability of data and materials:** Not applicable.
